# The neuropharmacology of the age-old sedative/hypnotic, ethanol

**DOI:** 10.3389/fnins.2013.00122

**Published:** 2013-07-15

**Authors:** Glenn W. Stevenson, Meredith A. Fox

**Affiliations:** ^1^Department of Psychology, University of New EnglandBiddeford, ME, USA; ^2^Laboratory of Clinical Science, National Institute of Mental Health, National Institutes of HealthBethesda, MD, USA

Ethyl alcohol (ethanol) is produced by the process of fermentation whereby plant sugars are converted to ethanol by yeast. Products of fermentation include mead from honey, wine from grapes, sake from rice, and beer from grains. Fermented products can also be distilled, for example when whisky is distilled from grains (Herbst and Herbst, 2003). The history of voluntary ethanol use by humans dates as far back as 8000 years ago for wine and possibly earlier for beer, as barley may have been the oldest cultivated grain (Pendell, 2010). It is also documented that ethanol was an extremely important staple to ancient civilizations such as the Egyptians and the Maya (Johnson, 1994), as well as more modern societies such as the original American colonies (Pendell, 2010). It seems that modern society's interest in alcohol has certainly not waned, and from the standpoint of neuropharmacology, has probably increased.

During the past several decades, there has been a concerted effort to determine the neuropharmacological mechanism of action(s) of ethanol. Ethanol has been reported to produce its effects via modulation of neural cell membrane fluidity as well as modulation of several neurotransmitter systems, including γ-amino butyric acid (GABA), glutamate, dopamine, and opioid systems (for reviews, see Koob et al., 1998; Kumar et al., 2009). GABA is an inhibitory amino acid neurotransmitter that is ubiquitously distributed in the mammalian brain, and ethanol's effects on the GABA system are thought to be mediated primarily by activating the GABA_A_ receptor, a 5-subunit receptor that gates Cl^−^ ions (for a review, see Kumar et al., 2009). Thus, GABA receptor activation produces CNS inhibition via Cl^−^ influx. It has also been reported that specific GABA_A_ receptor subunits mediate specific effects. For example, the α1 subunit is associated with sedation, whereas the α2 and α3 subunits are associated with anti-anxiety effects (Licata and Rowlett, 2008; Ator et al., 2010). Many types of sedative-hypnotic compounds (drugs producing dose-dependent sedation and ultimately sleep), including the benzodiazepines, barbiturates, and ethanol, bind to and activate the GABA_A_ receptor, and it is reasonable to assume that the sedative effects of these agents are mediated, at least in part, by the α1 subunit.

It is known that neurotransmitters, including GABA, bind to their receptors as “first messengers,” and initiate a complex cascade of intra-cellular events. In the case of GABA, part of this cellular cascade includes modulation of the “second messenger” systems Ca^++^ and cAMP that ultimately modulate two protein kinases, PKC and PKA, resulting in myriad effects in the cell, including changes to gene expression (Moss et al., 1992; Diamond and Gordon, 1997; Brandon et al., 2000 for a review, see Kumar et al., 2009).

Leslie Morrow and colleagues have been exploring the complex cascade of downstream cellular events mediated by ethanol-induced GABA_A_ receptor activation. In their recent Frontiers in Neuroscience report (Kumar et al., 2012), this research team determined the effects of acute ethanol exposure on PKA-mediated GABA_A_ receptor expression. Although chronic ethanol exposure has been reported to produce down-regulation and/or desensitization of GABA_A_ receptors, Morrow and colleagues reported an interesting finding of acute ethanol exposure. Specifically, acute intracerebroventricular administration of ethanol produced dose-dependent *increases* in expression of PKA and GABA_A_ receptor α1 subunits. In addition, antagonism studies revealed that blockade of PKA blocked the ethanol-induced increases in GABA_A_ receptor α1 subunit expression, providing further evidence for a PKA-GABA_A_ receptor network communication. An additional manipulation showed that enhancing PKA activity actually enhanced ethanol-induced loss of righting reflex. Together with earlier reports from this research group, these data indicate that PKA (Kumar et al., 2012) and PKC (Kumar et al., 2006) may have antagonistic effects on GABA_A_ receptor α1 subunit expression, thus delineating specific potential pathway(s) for mediation of ethanol effects on cell function and overt organismal behavior. This group's current report represents a comprehensive analysis of the specific effects of ethanol on kinase activity and receptor subunit expression levels, and adds another layer of knowledge about the neuropharmacological mechanisms of ethanol. It is quite possible that further elucidation of the complex molecular “systems level” analysis of ethanol's actions in nerve cells may yield important “systems level” approaches and advances for the treatment of alcohol abuse and dependence.
